# Observation of a comb-shaped filamentary plasma array under subcritical condition in 303-GHz millimetre-wave air discharge

**DOI:** 10.1038/s41598-019-54333-5

**Published:** 2019-11-29

**Authors:** Masafumi Fukunari, Shunsuke Tanaka, Ryuji Shinbayashi, Yuusuke Yamaguchi, Yoshinori Tatematsu, Teruo Saito

**Affiliations:** Research Center for Development of Far-Infrared Region, University of Fukui (FIR-UF), Bunkyo 3-9-1, Fukui, 910-8507 Japan

**Keywords:** Terahertz optics, Laser-produced plasmas

## Abstract

Gas breakdown in the millimetre-wave frequency band is an interesting phenomenon in nonlinear dynamics such as self-organized structure formation. We observed the transition between two types of filamentary plasma arrays in air discharge driven by a 303-GHz millimetre wave. Plasma is ignited at a parabolic mirror’s focal point in the overcritical condition. One array parallel to the electric field vector appears with a spacing of λ/4 at the focal point. Filaments then separate into plasma lumps ~10 μs after ignition. At 20 μs, a new comb-shaped array grows in the subcritical condition. Filaments are parallel to the incident beam with spacing of 0.96 λ and elongate towards the incident beam. This comb-shaped array appears only in the electric field plane; bulk plasma with a sharp vertex forms in the magnetic field plane. This array is created by a standing wave structure generated by waves diffracted from the plasma surface. Filamentary plasma array formations can influence the energy absorption by the plasma, which is important for engineering applications such as beamed energy propulsion.

## Introduction

Gas breakdown generated by a millimetre-wave beam has been widely studied for applications including beamed energy propulsion^1,2^, communication technology^3,4^, and ultraviolet (UV) radiation sources^5,6^. Gyrotrons are generally used as a high-power millimetre-wave source to ignite plasma^7,8^. In high-power millimetre-wave discharges, self-organized structures called as ‘filaments’ or ‘fish bones’ are formed^9^.

The discharge condition can be categorized as overcritical (OC) or subcritical (SC) depending on the ratio of the incident beam intensity to the ionization threshold, also called the critical intensity (e.g. Eq. (1.1) in ref. ^3^ or Eq. (8) in ref.  ^4^). Studies have investigated the formation of filamentary arrays with spacing of one-quarter of the wavelength λ (λ/4 structure) under OC condition, especially at a frequency of 110 GHz^10–12^. This condition is typically satisfied at the focal point of a lens or a mirror. In the E-k plane parallel to the electric field vector (E vector) and the wave vector, each plasma filament elongates along the electric field, whereas in the H-k plane parallel to the magnetic field vector (H vector) and the wave vector, a triangular lattice structure is observed^10–12^. The ionization front propagates towards the beam source with velocities of 10^4^ to 10^5^ m/s. Filamentary array formation has been qualitatively reproduced using numerical models^13–16^. Experimental and numerical studies indicate that their structure is determined by the diffusion process of electrons and by the standing wave generated by the incident beam and reflected beam from the ionization front.

Relatively few studies have investigated the SC condition. Time-integrated images obtained at 86 GHz in air^17^, 170 GHz in air^18^, 550 GHz in inert gases and nitrogen^19^, and 670 GHz in inert gases and nitrogen^6^ showed self-organized filaments. However the discharge structure was not the λ/4 structure. Furthermore, under this condition, the propagation velocity of the ionization front was on the order of 10^2^ m/s. The ionization model proposed for the OC condition does not hold for the SC condition. Voskoboinikova *et al*.^20^ proposed that the reduction of gas density due to Joule heating contributes to ionization. Takahashi *et al*.^21^ discussed structure formation with the ionization model. However, this model, in which the expansion wave is in front of the ionization front, has not yet been verified experimentally. Bogatov *et al*.^17^ measured the electron number density in a precursor (plasma halo) created at the ionization front by the UV radiation from the bulk plasma and indicated that the precursor supports ionization. In addition, they revealed that when the incident beam intensity is lower than a threshold, the ambient gas is heated to a temperature at which thermal ionization becomes effective. Because the discharge structure in this case is determined by the gas temperature distribution, filaments are not formed. Upon increasing the incident beam intensity, the discharge enters a thermal nonequilibrium state and the gas temperature decreases. The self-organized filamentary structure is thus in nonequilibrium and depends on the electric field distribution.

A comb-shaped filamentary plasma array parallel to the incident beam has been predicted using an artificial ionization term with low critical intensity^22^. Although the pitch of 0.9 λ between filaments agreed with the experiments at 170 GHz (ref. ^22^ shows fast-framing image data at 170 GHz), the comb-shaped array has not yet been observed.

The discharge structure in both the E-k and the H-k planes under the SC condition and its pressure dependence have been investigated at the relatively low frequencies of 15 GHz^2^ and 28 GHz^23^. In both cases, irregular sponge-like filaments were observed at a pressure close to atmospheric pressure. The structure was slightly different between the E-k and the H-k planes. For pressure lowers than around 20–30 kPa, the discharge structure became the λ/4 structure. Although the critical intensity decreases with pressure, the field intensity is still in the SC condition. Botanov *et al*.^2^ explained that the transition of the discharge structure was caused by the change in the mean free path of the ionization photon. The λ/4 structure formation under the SC condition near the OC condition has also been predicted from numerical computations at 170 GHz by Takahashi *et al*.^21^. Takahashi *et al*. explained that with increasing millimetre wave beam intensity, the reflectivity of the plasma increases due to the reduction of gas density and the reduced electric field intensity exceeds the critical intensity, resulting in the transition. The critical intensity is a function of the pressure (number density of the gas) and beam frequency. The reductions of the pressure and frequency lead to a decrease in critical intensity as far as plasma diffusion is not dominant. Thus, the transition occurs at low pressure and low frequency.

The transition of the discharge structure is an important process because in most cases, the discharge is ignited under the OC condition and propagates under the SC condition. However, the irregular sponge-like filaments imply a complex and different feature from that in high-frequency cases. To the best of our knowledge, no experimental study has investigated the transition in the high-frequency range.

Therefore, detailed experiments of millimetre-wave discharge under the SC condition are strongly required. Toward this end, in this study, we observed the whole dynamic process of millimetre-wave discharge in air at 303 GHz from the OC condition to the SC condition in both the E-k and the H-k planes using a high-speed shutter camera.

## Experimental Apparatus

**Figure 1:**
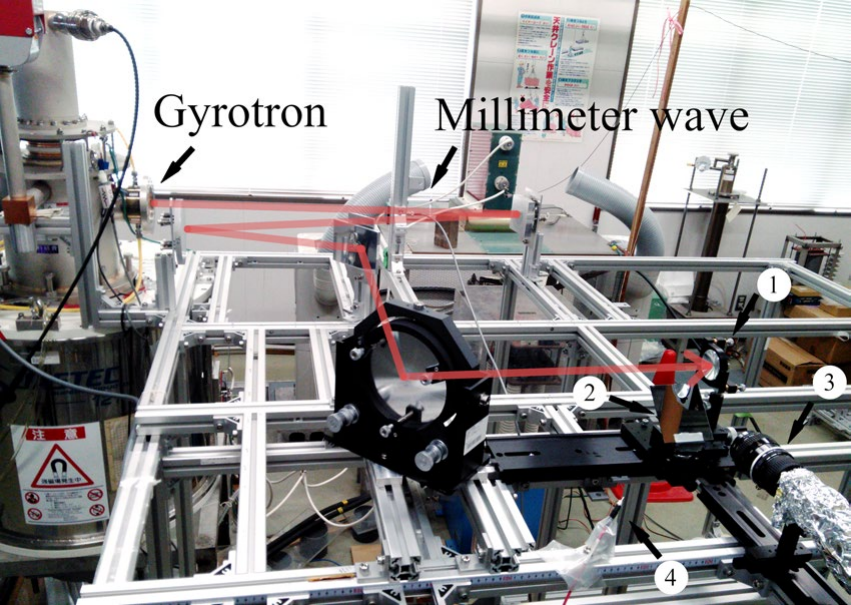
Experimental setup. 1: Parabolic mirror, 2: Flat mirror, 3: High-speed shutter camera, 4: Arc sensor.

Figure 1 shows the experimental setup. A 303-GHz high-power gyrotron was used as a beam source^24,25^. A parabolic mirror was used to ignite the discharge by focusing the incident beam. The high frequency of 303 GHz provides sharp focusing, and the OC condition is satisfied at the focal point. The SC condition holds along the incident beam. The diameter and focal length of the parabolic mirror were 50 mm and 18 mm, respectively. The E vector of the incident beam is in the horizontal direction. The discharge structure in the E-k plane was observed with a flat mirror installed under the focal point at an angle of 45°. The ignition timing was detected using an arc sensor. The radiation profile of the incident beam was an axisymmetric Gaussian beam. The incident beam power was measured to be 104 kW using a water dummy load.

The critical intensity *E*_cr_ differs slightly depending on the ionization model. In ref. ^3^, the critical intensity is described as

1 $$\begin{array}{rcl}{E}_{{\rm{cr}}} & = & 28.2{\rm{C}}({\nu }_{{\rm{c}}}/\omega )(\frac{{N}_{{\rm{m}}}}{2.7\times {10}^{19}{\mathrm{cm}}^{-3}}){(1+\frac{{\omega }^{2}}{{\nu }_{{\rm{c}}}^{2}})}^{1/2}\,[{\rm{kV}}\,{{\rm{cm}}}^{-1}]\\ {\nu }_{{\rm{c}}} & = & 1.7({N}_{{\rm{m}}}/{10}^{7}{\mathrm{cm}}^{-3})\,[{s}^{-1}]\end{array}$$

Here *N*_m_ is the number density of the air, *ν*_c_ is characteristic electron collision frequency, and C(ν_c_/*ω*) is a coefficient which equals to 1 for ω/ν_c_ ≪ 1 (In the case of 303 GHz atmospheric condition, ω/ν_c_ ≈ 0.47). At a pressure *p* = 760 torr and temperature T ~ 300 K, *N*_m_ = 2.7 × 10^19^ cm^−3^. This model does not include effects of the diffusion and represents the critical intensity in the high pressure region. At 303 GHz under atmospheric conditions, *E*_cr_ is estimated as 31 kV/cm as the amplitude of the incident electric field according to this model. In ref. ^4^, the critical intensity is

2 $$\begin{array}{rcl}{E}_{{\rm{cr}},{\rm{rms}}} & = & p{(1+\frac{{\omega }^{2}}{{\nu }_{{\rm{c}}}^{2}})}^{1/2}3.75{[(\frac{D}{p{\Lambda }^{2}})+6.4\times {10}^{4}]}^{3/16}\,[{{\rm{V}}\mathrm{cm}}^{-1}]\\ {\nu }_{{\rm{c}}} & = & 5.3\times {10}^{9}p\,[{s}^{-1}]\end{array}$$

Here *p* is the pressure in torr, *D* is the diffusion coefficient, and Λ is characteristic diffusion length. In ref. ^10^, Λ ≈ *w*_0_/*π* was used for the focusing beam radius *w*_0_ and this model was compared with experimental results. This model denotes the root mean square (RMS) field. Aassuming the free electron diffusion, the electron temperature of 2 eV, and the beam radius of λ at the focal point, the critical intensity is deduced as 37 kV/cm as the amplitude of the incident electric field.

The beam radius and radius of curvature of the incident beam at the parabolic mirror were estimated as 14.15 mm and 667.3 mm from radiation profile measurements with an infrared camera, respectively. The peak power density of the incident beam to the parabolic mirror was accordingly estimated as 33 kW/cm^2^, which corresponds to an intensity of 5.0 kV/cm as the oscillating electric field. Assuming the beam radius of λ at the focal point^26^, the average beam intensity reaches to 51 kV/cm. This value is safely larger than the critical intensities estimated above.

## Discharge Structure and Propagation Velocity of the Ionization Front

**Figure 2:**
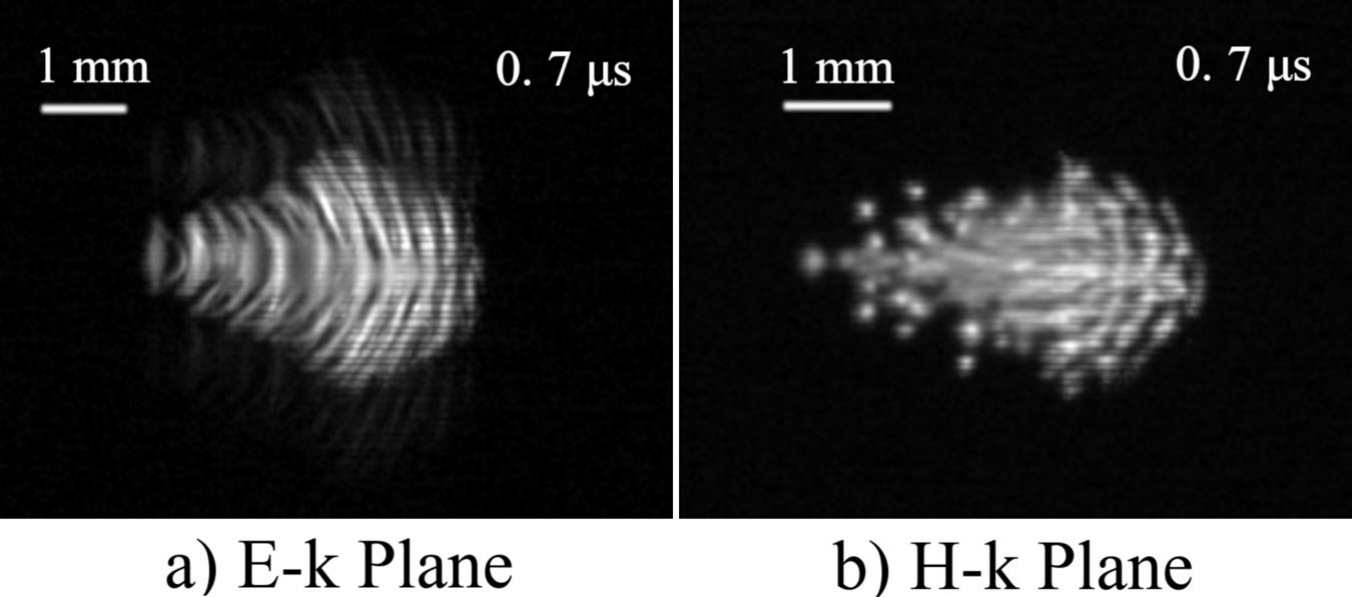
Discharge structure at focal point. Incident beam propagates from left to right. Parabolic mirror is set at right-hand side, and its edge is located 10.4 mm away from the focal point.

Figure 2 shows the discharge structure near the focal point. The incident beam propagates from left to right. The parabolic mirror is located at the right-hand side. The camera’s exposure time was set at 1 μs. The shutter timing was set earlier than the ignition to observe the beginning of the discharge. The ionization front propagates towards the parabolic mirror. Plasma filaments elongated along the E vector. The pitch between the filaments in the E-k plane was 0.26 λ on average; this was a typical λ/4 structure. We found that 303 GHz is the highest frequency at which the λ/4 structure is observed experimentally so far.

**Figure 3:**
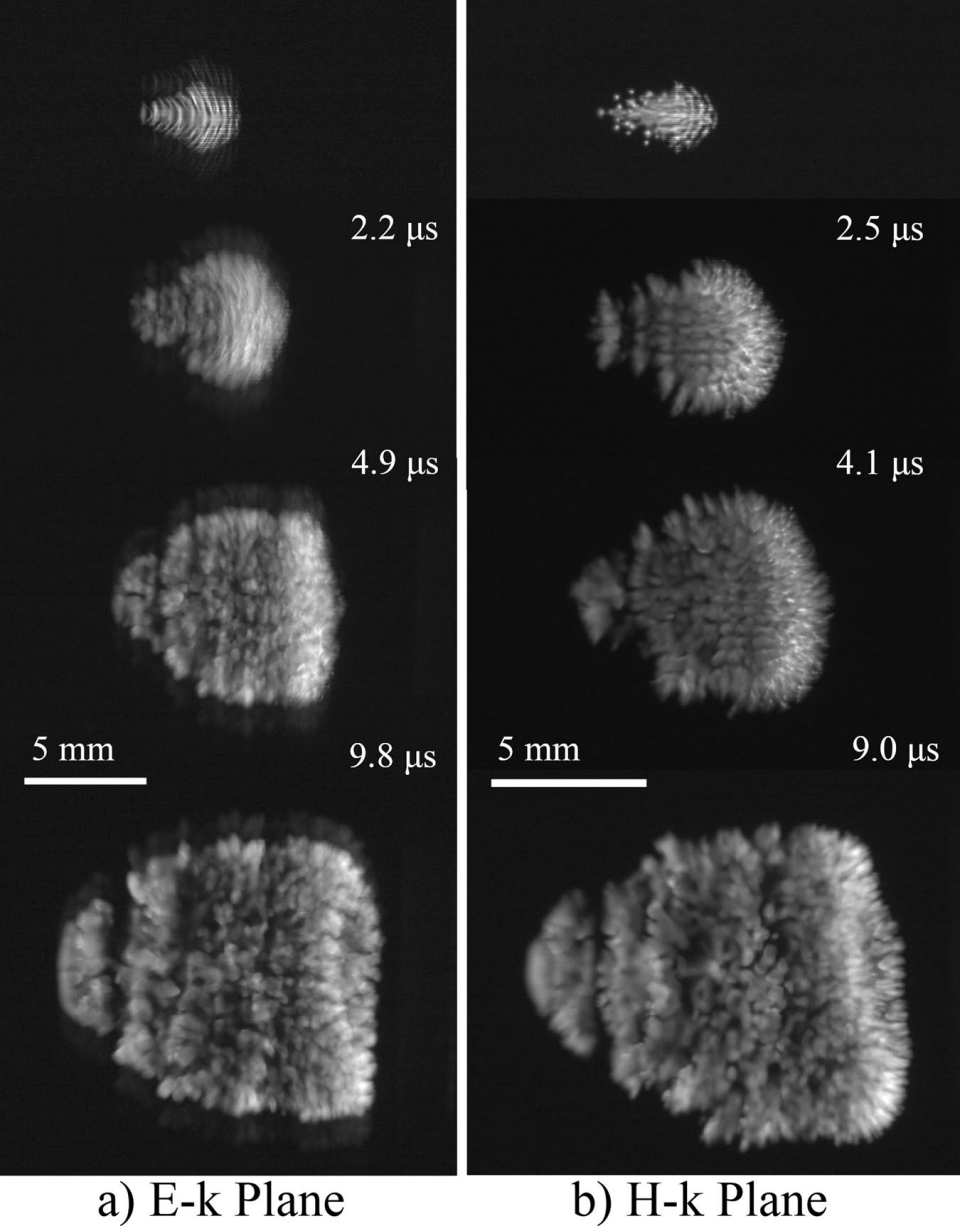
Transition of discharge structure from 2 to 10 μs. Incident beam propagates from left to right. Top images are the same as those in Fig. 2 at the same spatial scale.

After ignition, the filaments in the E-k plane gradually separated into lumps of plasma, as shown in Fig. 3. At 10 μs after ignition, the discharge structure became similar to that in the H-k plane. The diameters of the lumps were 0.2–0.8 λ.

**Figure 4:**
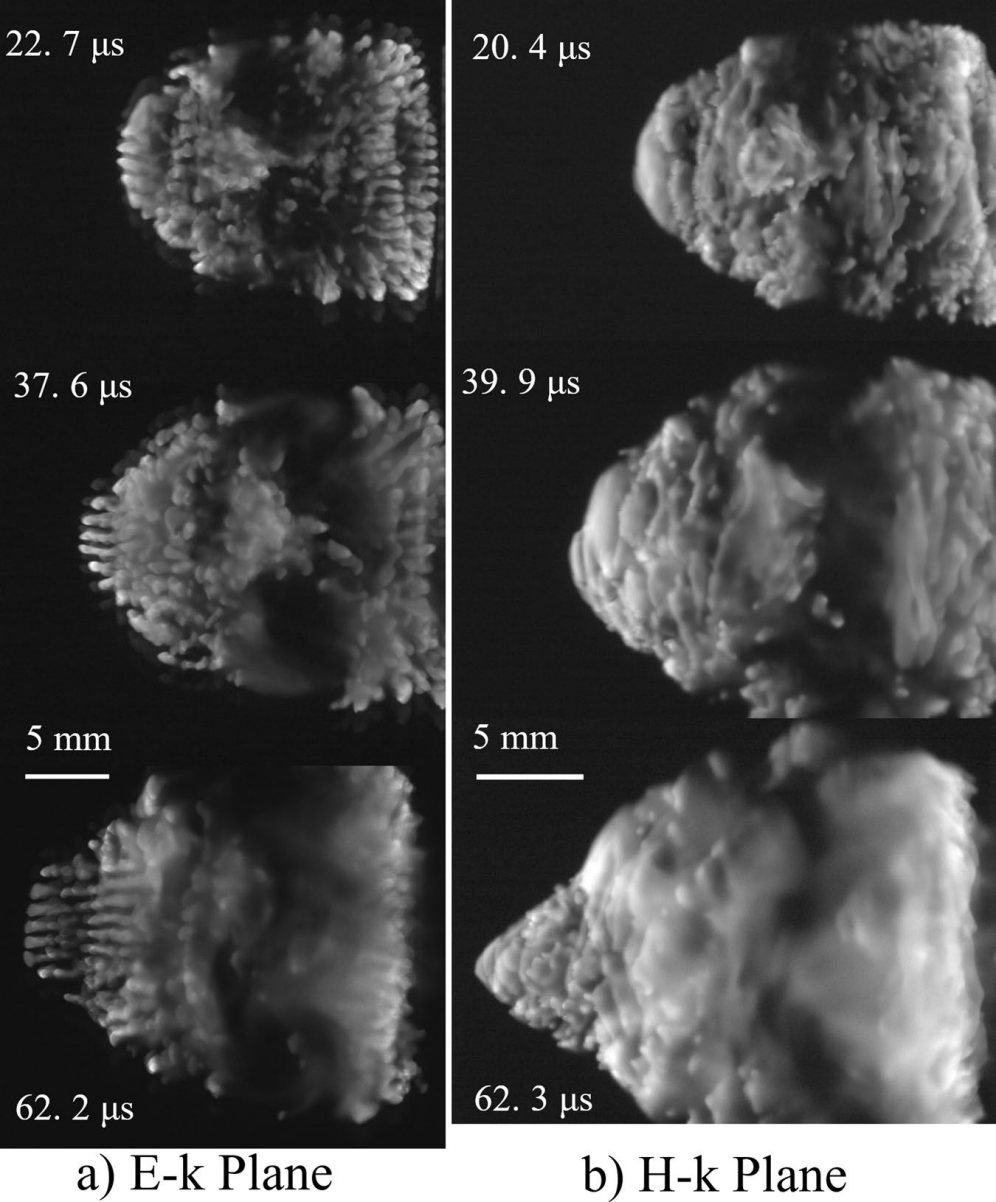
Transition of discharge structure from 20 to 60 μs. Incident beam propagates from left to right.

The ionization front at the left-hand side of the plasma begins to propagate towards the incident beam. This ionization front is under the SC condition. We observed the transition of structure formation from the OC condition to the SC condition. Figure 4 shows the discharge structures from 20 to 60 μs. At 22 μs, new plasma filaments parallel to the incident beam start to grow in the E-k plane. The pitch between each filament was estimated to be 0.96 λ on average. The width of the filament was 0.8 λ on average. The comb-shaped filamentary plasma array predicted in ref. ^22^ at 170 GHz was confirmed clearly in the experiments for the first time. In contrast, in the H-k plane, the plasma maintains a continuous structure and the ionization front forms a vertex at 60 μs. Note that filamentary array formation in the H-k plane was numerically predicted at 170 GHz in ref. ^21^.

**Figure 5:**
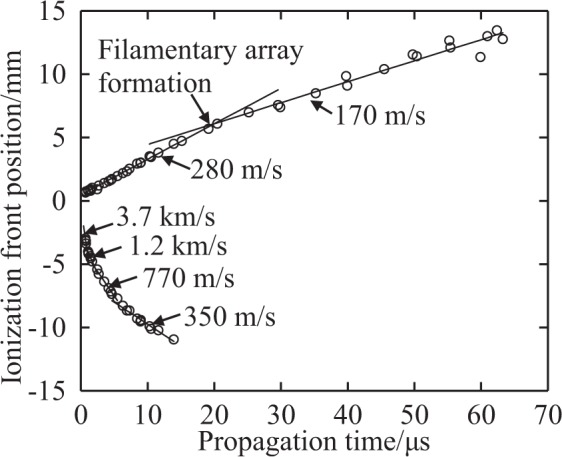
Ionization front position and its propagation velocity. Origin is set at the focal point(18 mm).

Figure 5 shows plots of the ionization front positions propagating to the parabolic mirror (lower plots) and backwards to the incident beam (upper plots) as functions of the time after ignition. The ionization fronts are the furthest edges on the right- and the left-hand sides. According to the estimated beam intensity at the ionization front position, the OC condition terminates at ~1 μs. The velocity of the ionization front propagating towards the parabolic mirror decreases gradually from 3.7 km/s to 350 m/s. This ionization front propagation is supported by the millimetre wave passing through the plasma side edge, which is also reflected by the parabolic mirror. The velocity of the ionization front propagating backwards to the incident beam is 280 m/s until ~20 μs and decreases to 170 m/s after 20 μs. At this time, the new comb-shaped filaments parallel to the incident beam begin to grow.

The decrease in propagation velocity when filament formation occurs is an unexpected result because, generally, the propagation velocity of the ionization front increases with the electric field intensity and the filaments cause electric field concentration. A possible mechanism for the decrease is that the electron temperature may decrease along the propagation under the SC condition, resulting in a decrease in the ionization frequency. In addition, shock wave detachment from the ionization front and the lateral expansion of the plasma can also influence the propagation velocity.

## Formation Mechanism of the Comb-Shaped Filamentary Plasma Array

The formation of the comb-shaped filamentary plasma array and the pitch between the filament agrees with the numerical prediction in ref. ^22^ except for the plasma width (0.5 λ in ref. ^22^). The pitch depends on the plasma width in the interpretation given in ref. ^22^. In addition, ref. ^15^. indicates that the plasma width is determined nonlinearly by the incident beam intensity and electron density. Therefore, the structure formation mechanism should be reconsidered. ref. ^17^. and ref. ^22^. suggest that the filamentary structure is determined by the wave-field distribution. Thus, we consider a simple model of wave diffraction from the fixed plasma distribution to investigate the formation of the comb-shaped filamentary plasma array, as shown in Fig. 4. The Kirchhoff diffraction formula is written as

3 $$U(P)=-\,\frac{1}{4\pi }\mathop{\oint }\limits_{S}\,d\tilde{s}\{U(S)(\frac{\exp (i2\pi \tilde{r})}{\tilde{r}})[i2\pi -(i2\pi -\frac{1}{\tilde{r}})\cos \,\theta ]\}$$

**Figure 6:**
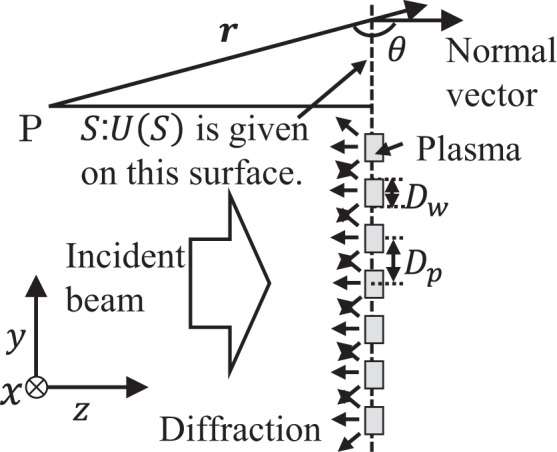
Configuration of the calculation.

Figure 6 shows the configuration of the calculation, where *r* and *θ* denote the distance from a point on the surface *S* to the observation point *P* and angle of the *r* vector to the normal vector of *S*, respectively. In Eq. (3), the spatial scales *r* and *s* are normalized by λ to $\tilde{r}$ and $\tilde{s}$. The wave number, *k* then becomes 2*π*. The scalar wave field at *P*, *U*(*P*), is calculated with a given scalar wave field distribution on *S*, *U*(*S*), a plasma width *D*_w_, and a pitch *D*_p_.

**Figure 7:**
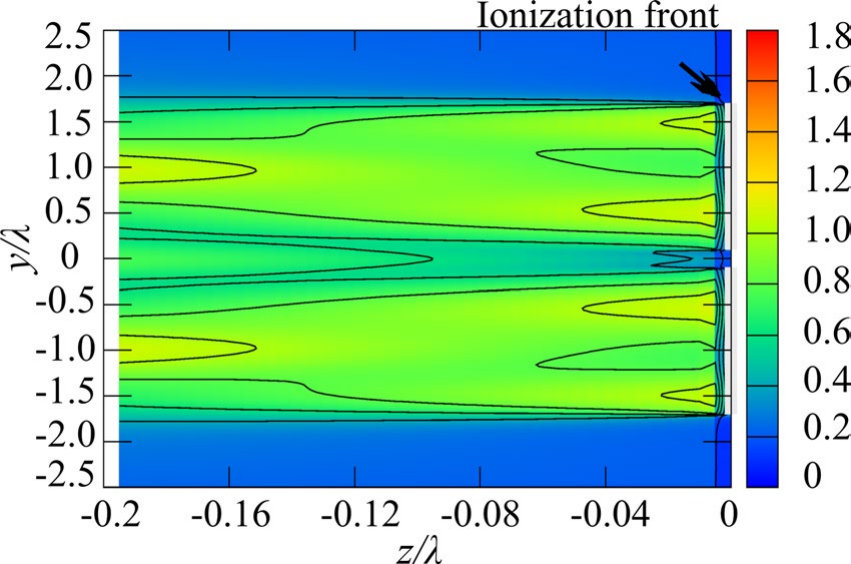
Calculation results of wave-field for two plasmas. *D*_w_ = 1.6λ, *D*_p_ = 1.8λ.

According to numerical computation results under the OC condition in ref. ^15^, when the electron number density is no longer negligible, the plasma behaves as a conductor and the electric field at the filament edge is enhanced. Therefore, the decay of the electron number density occurs at the centre of the filament. In Fig. 3a, at 2.2 μs and at 4.9 μs, the filament at the left-hand side that faces the incident beam splits into two. Next, we consider the situation in Fig. 3a at 4.9 μs, that is, *D*_p_ = 1.8λ and *D*_w_ = 1.6λ for two plasmas. The amplitude of *U*(*S*) was set as 1 in the plasma regions and 0 in the other regions. The plasma length in the *x* direction is set as 2λ. This value has a very weak effect. Figure 7 shows the diffracted wave intensity without the incident beam. The interference of diffracted waves creates the electric field concentration on the plasma surface with spacing of λ. Therefore, the comb-shaped filamentary array parallel to the wave vector begins to grow.

**Figure 8:**
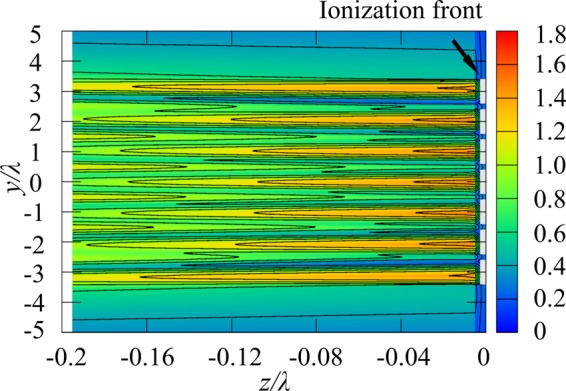
Calculation results of wave-field for seven plasmas. *D*_w_ = 0.8λ, *D*_p_ = λ.

Figure 8 shows calculation results of the diffracted wave field for *D*_p_ = λ and *D*_w_ = 0.8λ for seven plasmas corresponding to the experimental result in Fig. 4a. After filament formation, the diffracted waves propagate in both the *z*- and the *y*-directions and form a standing wave structure along the *y*-direction. Under the OC condition, the standing wave structure along the z direction creates the λ/4 structure. The peaks of the wave field in Fig. 8 are generated at the plasma front, and therefore, the ionization front can propagate along this structure. The standing wave under the SC condition is generated independently of *D*_w_ when the plasma structure has *D*_p_ close to λ. Although peaks with relatively low intensity are also generated between the plasma filaments (0.5 λ pitch) in Fig. 8, especially for small plasma width, the plasma does not grow due to the low plasma density. The curvature of the ionization front that is neglected in the analysis may cause the standing wave to expand and, in turn, the filaments, as shown in previous experimental researches.

In this study, the gas temperature was not measured. According to Bogatov *et al*.^17^, the gas temperature under nonequilibrium discharge was estimated to be 1500–1800 K. Although the reduction in gas density due to Joule heating and the precursor created at the ionization front by UV radiation support ionization, the discharge structure is determined by the electric field distribution as far as thermal ionization is not dominant. The standing wave thus determines the pitch under SC condition independently of *D*_w_.

## Conclusion

The dynamic transition of the discharge structure from OC to SC conditions has been observed as a series of images. In particular, a numerically predicted comb-shaped filamentary plasma array was clearly confirmed under SC condition for the first time. In addition, the difference in plasma structures in the E-k and the H-k planes under SC condition was found for the first time. This difference was not obtained in the numerical calculation for 170 GHz. This structure is created by a standing wave generated by waves diffracted from the plasma surface.

Filamentary plasma array formations can influence the energy absorption by the plasma. In particular, a comb-shaped filamentary plasma array works as a diffraction grating. This effect is important for the previously mentioned engineering applications. Understanding the formation mechanism of filamentary arrays is important for estimating reflection from the plasma.

## Data Availability

The datasets generated during and analysed during the current study are available from the corresponding author on reasonable request.
